# Holotomography and Multivariate Analysis Reveal Donor-Specific Responses to Antioxidant Supplementation During Stallion Sperm Cryopreservation

**DOI:** 10.3390/antiox15050642

**Published:** 2026-05-18

**Authors:** Graziano Preziosi, Raffaele Boni, Stefano Cecchini Gualandi, Maria Antonietta Ferrara

**Affiliations:** 1 Institute of Applied Sciences and Intelligent Systems, Unit of Naples, Italian National Research Council (ISASI-CNR), Via Pietro Castellino 111, 80131 Napoli, Italy; graziano.preziosi@na.isasi.cnr.it; 2 Department of Basic and Applied Sciences, University of Basilicata, Via dell’Ateneo Lucano 10, 85100 Potenza, Italy; raffaele.boni@unibas.it (R.B.); stefano.cecchini@unibas.it (S.C.G.)

**Keywords:** holotomography, quantitative imaging, stallion semen, antioxidant extracts, matcha, spirulina, horseradish, quercetin

## Abstract

Freeze–thaw procedures impair sperm morphology and function, affecting viability, motility, redox balance, and subcellular organization. Although antioxidants may mitigate these effects, their interaction with donor-specific variability remains unclear. We combined quantitative holotomography with conventional physiological assessments within a multivariate framework based on principal component analysis (PCA) and nested cross-validated Linear Discriminant Analysis (LDA) to evaluate donor-specific responses to antioxidant-supplemented cryopreservation. Spermatozoa from ten stallions was analyzed before and after freezing under five conditions: fresh semen; frozen semen with INRA Freeze, frozen semen with HF-20, and HF-20 supplemented with matcha, spirulina, horseradish, or quercetin. For each condition, sperm kinetics, mitochondrial activity, oxidative stress, DNA integrity, and three-dimensional volumetric measurements of whole-cell and subcellular compartments derived from holotomography were integrated into a single dataset. LDA achieved 0.734 cross-validated accuracy for stallion classification, revealing strong donor-specific signatures. In contrast, classification by antioxidant treatment was near chance (0.248). Fresh semen was clearly distinct from all cryopreserved groups. Holotomography showed reduced whole-cell and post-acrosomal/midpiece volumes after freezing, while nuclear volume was unchanged. Antioxidant supplementation produced minor, inconsistent effects, with partial midpiece preservation in some donors but no global pattern. Overall, inter-stallion variability dominates post-thaw sperm phenotype. Antioxidant effects were detectable but modest, supporting individualized strategies to optimize equine semen cryopreservation protocols.

## 1. Introduction

The long-term storage of equine genetic material is pivotal to modern breeding programs, facilitating assisted reproduction technologies and the global dissemination of valuable genetic traits [1,2]. Nevertheless, the freezing-thawing process imposes substantial physical and biochemical stress on spermatozoa, often resulting in reduced post-thaw quality and marked variability in semen performance [3,4].

Freeze–thaw procedures are known to impair sperm viability and motility [5], mitochondrial activity [6], and redox balance [7], while also inducing subtle yet significant alterations in sperm morphology and subcellular organization [8]. These effects are highly variable among individual stallions [9] and are influenced by both intrinsic biological factors and the composition of freezing extenders [10,11,12].

In recent years, antioxidant supplementation has been proposed as an effective strategy to mitigate cryo-induced oxidative damages and preserve sperm function [13]. Natural antioxidant extracts, such as plant- and algae-derived compounds, have attracted increasing attention due to their ability to modulate reactive oxygen species while supporting mitochondrial function and membrane integrity [14,15]. Nonetheless, the biological impact of different antioxidants on equine spermatozoa remains unclear, particularly when evaluated at the level of sperm subcellular structure and in relation to inter-individual variability among donors.

Beyond conventional semen analysis, advanced label-free imaging techniques offer new opportunities to investigate cryo-induced sperm damage. Holotomographic microscopy (Tomocube, Daejeon, Republic of Korea) provides three-dimensional refractive index (RI) maps of individual spermatozoa, enabling quantitative, non-invasive assessment of whole-cell and subcellular volumes [16,17]. This approach allows for the detection of structural alterations that are not readily captured by standard morphological or functional assays and offers a powerful complement to physiological-based evaluations. In a previous study [15], we first tested the application of holotomography (HT) in evaluating sperm quality in stallion. Examining the results obtained, we attributed a better discriminant capacity between the study groups to the sperm midpiece compared to other evaluated parameters such as the whole cell and the nuclear region. Analysis of the sperm midpiece was able to markedly discriminate between fresh and frozen spermatozoa but proved unable to provide comparative indications regarding the effects resulting from the use of different freezing extenders.

Given the multidimensional nature of sperm quality, multivariate statistical approaches are increasingly required to integrate heterogeneous datasets and extract biologically meaningful patterns [18,19,20]. Techniques such as principal component analysis (PCA) and supervised classification models allow the simultaneous evaluation of kinematic, oxidative, DNA fragmentation and structural parameters, facilitating the identification of key features driving sample variability and classification performance. Importantly, these approaches also enable the assessment of donor-dependent responses to cryopreservation and antioxidant treatments.

In the present study, semen samples treated with selected antioxidant compounds were analyzed using multiple assessment approaches to comprehensively assess the impact of cryopreservation and antioxidant supplementation. Specifically, spermatozoa were evaluated fresh, after cryopreservation with a commercial freezing extender (INRA Freeze), after cryopreservation with a freezing extender of known composition (HF-20), and after cryopreservation with HF-20 supplemented with natural antioxidants. The antioxidants tested included matcha, spirulina, horseradish, and quercetin, selected for their reported redox-modulating and cytoprotective properties [21,22,23,24]. Briefly, matcha, a green tea from *Camellia sinensis*, is rich in catechins, theanine, and caffeine [25]. Its effects on sperm cryopreservation are inconsistent, with benefits reported in rams, bulls, and humans [26,27,28], but not in pigs [23]. *Spirulina platensis*, a filamentous cyanobacterium, provides proteins, vitamins, minerals, fatty acids, and antioxidant pigments, support both enzymatic and non-enzymatic defense systems [29]. Its inclusion in freezing media has improved sperm motility and viability in buffalo bulls [22] and reduced cryodamage while enhancing post-thaw quality in stallions [30]. Horseradish (*Armoracia rusticana*) contains antioxidant compounds such as glucosinolates, flavonoids, and vitamins, though its application in sperm cryopreservation remains unexplored [31]. It is sometimes confused with *Moringa oleifera*, which has documented protective effects during sperm freezing [32]. Quercetin, a plant-derived flavonoid, serves as a reference antioxidant due to its ability to reduce oxidative stress and lipid peroxidation [33]. However, while it may modulate sperm metabolism [34], its effectiveness in preventing cryodamage remains uncertain [35].

We combined quantitative holotomographic analysis with multivariate statistical modeling to investigate the effects of cryopreservation and antioxidant supplementation on equine spermatozoa. By integrating sperm kinetics and selected sperm physiological parameters related to bioenergetics and oxidative/nitrosative stress markers with volumetric measurements of the whole cell and specific subcellular regions, we aimed to (i) characterize cryo-induced structural and functional alterations across fresh and frozen conditions; (ii) evaluate the protective effects of four considered natural antioxidant compounds added to a semi-defined freezing extender; and (iii) assess the relative contribution of donor identity and treatment condition to the observed variability.

Within this framework, our HT approach revealed marked cryopreservation-associated reductions in whole-cell and midpiece/post-acrosomal volumes, while nuclear volume remained largely preserved. Moreover, although certain antioxidants partially mitigated volumetric alterations, inter-stallion variability emerged as the dominant source of phenotypic variation, underscoring the need for individualized approaches in the optimization of equine semen cryopreservation protocols.

## 2. Materials and Methods

### 2.1. Sample Collection and Preparation

Between February and May 2025, semen samples were collected on a weekly basis from 10 clinically healthy and fertile Salernitano stallions, identified as donors #3 to #12 (age range: 4–17 years). Animals were individually housed in paddocks at the Regional Center for Equine Genetic Improvement (Caserta, Italy) under controlled and standardized management conditions. Stallions were fed a conventional diet consisting of hay and commercial concentrate, had free access to water, and did not receive any dietary antioxidant supplementation during the study period.

The semen collection facility is officially authorized by the Campania Regional Authority (authorization no. U1500083 CE000642004) and operates in compliance with current regulations and operational protocols concerning health, biosecurity, and animal welfare. All experimental procedures were conducted in accordance with European Directive 2010/63/EU and Italian legislation (D. Lgs. 26/2014), with particular attention to minimizing animal stress and reducing the number of animals used.

For each stallion, the collected ejaculate was divided into seven aliquots, generating one fresh sample and six samples subjected to different cryopreservation conditions. Specifically, for each individual donor, the following experimental groups were obtained: CTRL−, consisting of semen frozen using the HF-20 extender without antioxidant supplementation; extracts of Matcha (10 µg mL^−1^), Spirulina (5 µg mL^−1^), Horseradish (5 µg mL^−1^), and Quercetin (5 µg mL^−1^), in which these natural compounds were added as antioxidant supplements to semen frozen with the HF-20 extender; CTRL+, consisting of semen frozen using INRA Freeze, a commercially available freezing extender; and Fresh, representing non-frozen semen.

This experimental design enabled direct intra-donor comparisons across treatments while accounting for inter-individual variability. Detailed protocols for semen processing, antioxidant extract preparation, freezing procedures, and post-thaw handling are reported in [36].

### 2.2. Sperm Kinetics, Bioenergetics, and Oxidative/Nitrosative Stress Markers

Sperm motility was evaluated as previously described [25] using a computer-assisted sperm analyzer (SCA 5.0; Microptic, Barcellona, Spain). The following parameters were analyzed: total motility (TM), progressive motility (PM), curvilinear velocity (VCL), straight-line velocity (VSL), and average path velocity (VAP). Mitochondrial membrane potential (MMP), lipid peroxidation (LPO), intracellular reactive oxygen species (ROS), and nitric oxide (NO) levels were assessed as previously reported [36] using the fluorescent probes JC-1, C11-BODIPY^581/591^, H_2_DCFDA, and DAF-FM diacetate (Merck Life Science, Milan, Italy), respectively. Fluorescence signals were measured by fluorescence spectroscopy.

### 2.3. DNA Fragmentation Index

The DNA Fragmentation Index (DFI) was evaluated using both a direct method (the terminal deoxynucleotidyl transferase (TdT) dUTP Nick-End Labeling—APO-BrdU TUNEL Assay, TUNEL) and an indirect method (the sperm chromatin structure assay, SCSA) [36]. The TUNEL assay detects fragmented DNA by enzymatically labeling exposed 3′-OH ends with the thymidine analog 5-bromo-2′-deoxyuridine 5′-triphosphate (BrdUTP). SCSA measures the susceptibility of chromatin to denaturation after acid treatment and is based on the metachromatic properties of acridine orange (AO) in discriminating between intact double-stranded DNA and denatured single-stranded DNA. Details on the execution of these techniques and the results obtained are presented in a previous publication [36].

### 2.4. Holographic Tomography

Equine spermatozoa were imaged enabling a commercial high resolution holo-tomographic microscope (HT-2H) from Tomocube^®^ (Daejeon, Republic of Korea). It provides high-resolution 3D RI holo-tomograms of cells, facilitating the assessment of morphological details. The setup employs a Mach-Zehnder type interferometric configuration powered by a 532 nm light laser-emitting source. The optical field transmitted by the sample is captured using a high–numerical aperture 60× objective designed for water immersion. A digital micromirror device (DMD) is used to modulate the illumination direction, enabling the sequential acquisition of multiple 2D holograms at distinct angles. These angularly diverse datasets are processed to reconstruct a 3D refractive index map. The optical performance of the system yields spatial resolutions of approximately 110 nm laterally and 220 nm axially, supporting high-resolution biological imaging. An aliquot of 60 µL of each collected sample was placed into a dedicated Petri dish (Tomodish^®^, Daejeon, Republic of Korea) equipped with a small square glass window at the bottom to ensure minimal optical distortion and placed on a platform between the objective and condenser lens. A total of 30 sperm RI holo-tomograms were acquired for each analyzed sample. The obtained RI holo-tomograms were processed by using TomoAnalysis Classic^®^ software (version 1.3.29). It allowed for RI Thresholding analysis on the acquired images, applying deep learning techniques for sperm cell segmentation and the quantification of biophysical structural parameters. Focusing on volume, a comparison between fresh and frozen–thawed samples treated with different antioxidant extracts was performed.

### 2.5. Statistical Analysis

Statistical analyses were performed using JASP (version 0.19.3.0), an open-source program supported by the University of Amsterdam [37]. Data obtained from the HT assessment were processed by analysis of variance (ANOVA). Data related to sperm kinetics, mitochondrial activity, oxidative/nitrosative stress markers, and DNA damage have been not included, as they had been previously analyzed [36]. Percentage data were arcsine-transformed before the analysis. The Shapiro-Wilks test and Levene’s test were used to assess normality of data distribution and homogeneity of variance, respectively. Pairwise comparisons of means were performed using the Bonferroni post hoc test. Statistical significance was set at *p* < 0.05.

#### Multivariate Analysis

Multivariate classification of sperm samples by Stallion and Treatment was performed using Linear Discriminant Analysis (LDA), Support Vector Machines (SVM), and k-Nearest Neighbors (kNN). Prior to modelling, dimensionality reduction was applied via PCA. The dataset used for multivariate analysis was constructed by defining a feature vector for each experimental observation. Each vector included the donor identifier (10 donors in total), the type of treatment (Fresh, CTRL−, CTRL+, Matcha, Spirulina, Horseradish, and Quercetin), and the donor’s age. In addition, the feature vector incorporated multiple quantitative parameters organized into functional categories: (i) kinematic parameters describing sperm motility included TM, PM, VCL, VSL, and VAP; (ii) sperm bioenergetics included MMP; (iii) oxidative/nitrosative stress-related markers included LPO, ROS, and NO; (iv) DNA integrity indices assessed by TUNEL and SCSA; (v) volumetric parameters (volume of the whole cell, midpiece, and nucleus).

PCA was embedded within a nested 5-fold cross-validation (CV) framework (both outer and inner loops). The 5-fold CV scheme was selected to provide a stable compromise between bias and variance in performance estimation, while maintaining adequate sample representation within each training and test partition given the dataset size [38]. In the inner loop, the number of principal components (PCs, tested from 2 to 15) was optimized to maximize classification accuracy. Across outer folds, each fold’s inner loop identified an optimal number of PCs, and the median of these values was used to summarize the overall selection for each model. Feature matrices were standardized (z-score) prior to PCA. LDA (linear discriminant) was selected as the primary classifier due to its superior performance and robustness compared to SVM (RBF kernel) and kNN (k = 5). Nested cross-validated accuracy across outer folds was the main performance metric [39], and comparisons between models were visualized with bar plots of nested CV accuracies (see Figure S1). Although the median optimal number of PCs was 10 for Stallion (range 10–11) and 7 for Extract (range 3–14), the number of PCs was fixed at 5 for all LDA analyses to reduce model complexity and improve interpretability while still capturing >70% of the total variance. All analyses were implemented in a custom MATLAB code developed for this study (MATLAB version R_2024b).

## 3. Results

**Figure 1:**
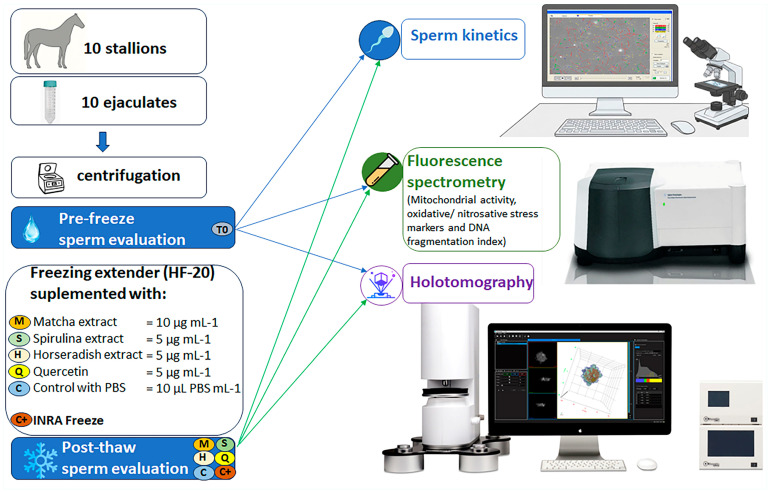
Schematic representation of the experimental design used to evaluate the cryoprotective effects of three natural extracts (matcha, spirulina, and horseradish) and quercetin on stallion sperm.

The results presented in this section were obtained combining sperm kinetics, bioenergetics, oxidative/nitrosative stress markers, and DNA fragmentation indices with refractive index imaging, volumetric analysis across specific sperm regions under different antioxidant treatments, and multivariate classification based on principal component analysis combined with linear discriminant analysis to evaluate structural variations and classification performance. A schematic representation of the experimental design is reported in Figure 1.

### 3.1. Holographic Tomography Analysis

**Figure 2:**
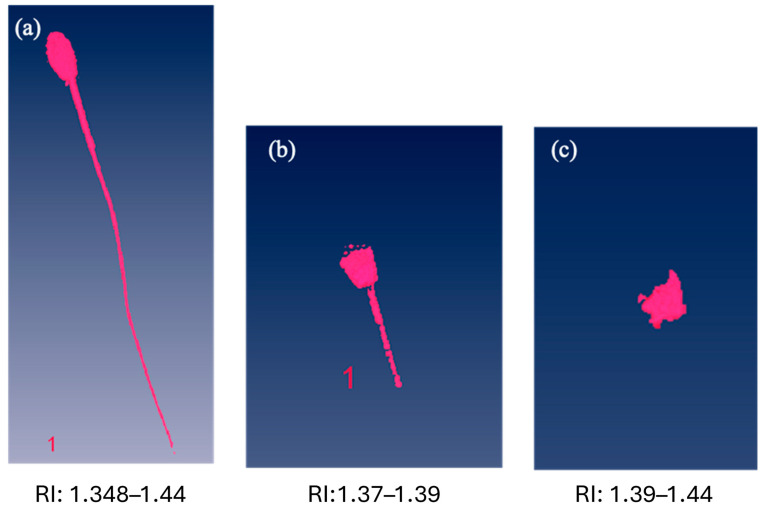
Micrographs of stallion spermatozoon obtained using RI acquisition, showing (**a**) whole cell, (**b**) post-acrosomal region and midpiece, (**c**) nuclear region. The corresponding RI ranges are reported alongside each figure.

Three-dimensional RI tomograms of equine spermatozoa were obtained via holotomographic analysis. These tomograms represent the spatial distribution of the sperm’s RI and were used to perform a quantitative analysis of the entire sperm structure as well as its subcellular components. Based on the specific RI ranges identified and validated in our earlier work [15], we characterized the whole sperm cell (Figure 2a), the post-acrosomal and mid-piece regions (Figure 2b), and the nuclear region (Figure 2c) by applying the same RI ranges to all analyzed sperm cells, including refrigerated and frozen–thawed samples either with or without natural antioxidant compounds such as matcha, spirulina, horseradish, and quercetin.

From the processed holograms, structural parameters were extracted to conduct quantitative comparisons across sperm regions and sample conditions. Among the quantified parameters, we focused primarily on volume, as it proved to be the most sensitive indicator of post-freezing changes in the spermatozoa.

**Figure 3:**
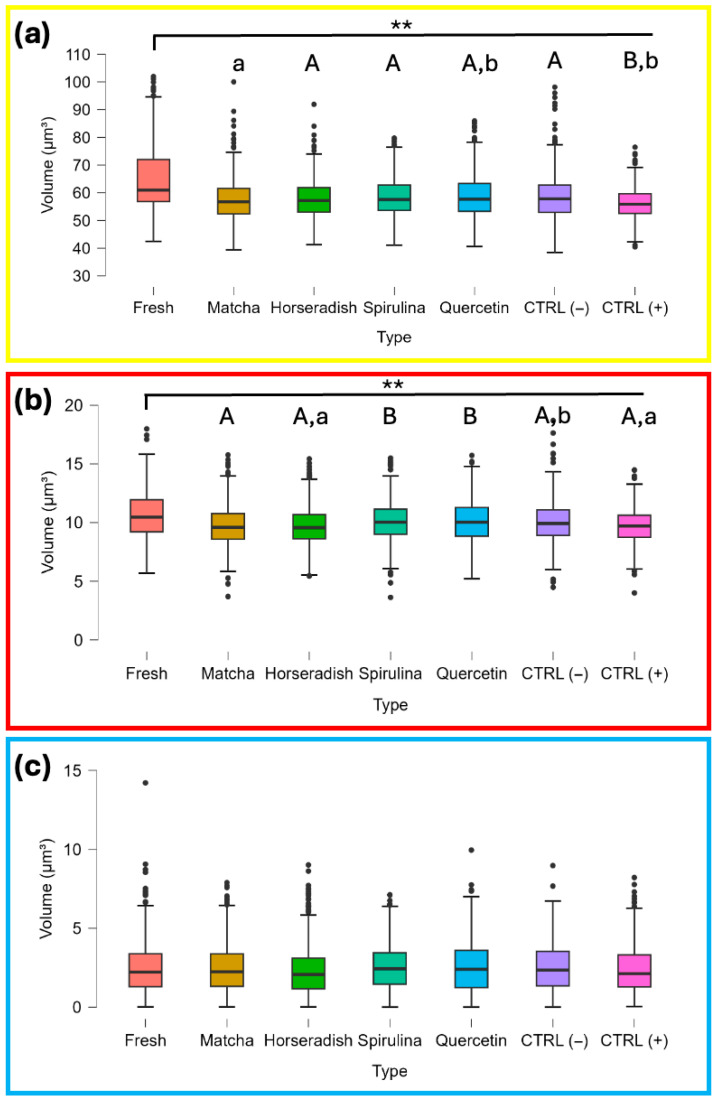
Assessment of sperm volume performed for whole cell region (**a**), post-acrosomal and midpiece region (**b**) and nuclear region (**c**) corresponding to different refractive index ranges. The box plots show antioxidant-related variations in fresh and frozen/thawed sperm, independent of individual variation. Bonferroni post hoc test was performed for statistical significance: a, b (*p* ≤ 0.05); A, B (*p* ≤ 0.01); ** (*p* < 0.01). Different colored boxes are used only to visually distinguish the different treatment conditions.

Thus, we investigated the effects of four antioxidant treatments on the volumes changes in specific sperm structural regions following cryopreservation. Figure 3 presents box plots showing the distribution of volume measurements for the three analyzed regions—whole cell (Figure 3a), mid-piece (Figure 3b), and nuclear region (Figure 3c)—according to the predefined RI ranges (Figure S2). The analysis included both fresh and frozen spermatozoa from all donors exposed to the different antioxidant compounds. Two control groups were also considered: a negative control (CTRL−), cryopreserved with HF-20, and a positive control (CTRL+), cryopreserved with INRA Freeze.

Holotomographic analysis revealed a reduction in sperm volume in frozen stallion samples affecting the whole sperm structure and the post-acrosomal region including the mid-piece, compared to fresh sperm. In contrast, the nuclear region volume of frozen equine spermatozoa did not differ significantly from that of fresh sperm.

Among the antioxidant treatments, matcha and horseradish showed a greater reduction in the volume of the post-acrosomal region, including the mid-piece, compared to spirulina and quercetin (*p* < 0.01) and CTRL− (*p* < 0.05) (Figure S3). The volume of this region was also significantly reduced in CTRL+ spermatozoa, frozen in INRA Freeze, compared to the groups supplemented with spirulina and quercetin (*p* < 0.01), as well as in CTRL− spermatozoa, frozen in HF-20 and not supplemented with antioxidants. Minor variations were observed in relation to the whole cell volume, with all groups treated with antioxidant substances, as well as CTRL−, showing significant differences compared to CTRL+. A slight difference (*p* < 0.05) also emerged between spermatozoa treated with matcha and quercetin. In contrast, the volumes of nuclear region remained relatively stable across frozen sperm samples under different antioxidant treatments.

**Figure 4:**
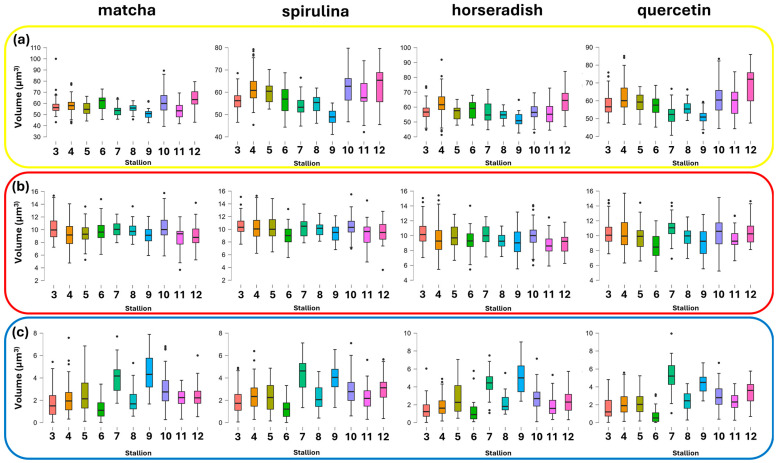
Volume of (**a**) whole cell, (**b**) post-acrosomal region and midpiece, (**c**) nuclear region are reported for each stallion across the different antioxidant treatments. Different colored boxes indicate different donors.

Furthermore, frozen sperm were analyzed by comparing individual donors within each antioxidant treatment (Figure 4) across all regions of interest. The box plots reveal marked inter-individual variability (*p* < 0.001) in response to semen cryopreservation, which was donor-dependent across the entire sperm structure (Figure 4a), the mid-piece (Figure 4b), and the nuclear region (Figure 4c).

### 3.2. Multivariate Analysis and Classification Performance

**Figure 5:**
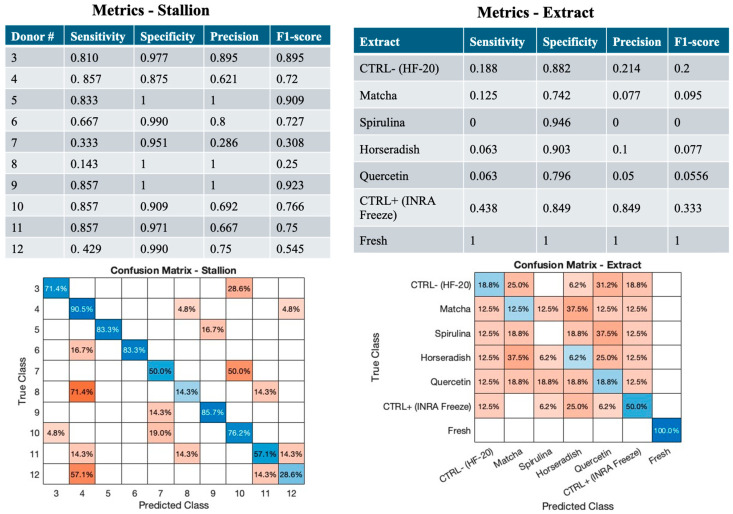
Multivariate classification performance based on principal component analysis (PCA) using five principal components (PCs); blue shades represent values along the diagonal, whereas red shades represent off-diagonal values; darker shades indicate higher values and lighter shades indicate lower values. On the left, stallion-based classification results are shown, with the upper panel reporting class-wise performance metrics (Sensitivity, Specificity, Precision, F1-score) and the lower panel displaying the corresponding confusion matrix. On the right, extract-based classification results are presented, with the upper panel summarizing the same performance metrics and the lower panel showing the confusion matrix obtained using five PCs. Sensitivity represents the proportion of samples from a given class that are correctly identified by the classifier, whereas Specificity indicates the proportion of samples not belonging to a given class that are correctly classified as not belonging to that class. Precision reflects the proportion of samples predicted as belonging to a given class that truly belong to that class. The F1-score, defined as the harmonic mean of precision and sensitivity, provides a balanced measure of classification performance, particularly in the presence of class imbalance. Performance metrics were computed as described in Equation (A1) in Appendix A.

All the evaluated features of equine spermatozoa were analyzed via PCA to reduce dimensionality and capture major sources of variance. The complete set of sperm quality parameters, including sperm kinetics, bioenergetics, oxidative and nitrosative stress markers, and DNA fragmentation indices, is presented in Figure S4; these variables were directly incorporated into the multivariate dataset used for PCA and LDA analyses. The first five principal components were retained for classification. LDA was applied to classify samples according to stallion identity and antioxidant treatment. Classification performance was evaluated using nested cross-validation, which integrates PCA within each fold to prevent information leakage. Metrics including accuracy, precision, sensitivity, specificity and F1-score were calculated for each class, as described in Equation (A1) in Appendix A [40,41,42]. Confusion matrices were also generated to visualize prediction performance. Results are summarized in Figure 5, while PCA loadings is reported in Figure S5.

When samples were classified according to Stallion identity (10 classes), the model achieved a nested cross-validated accuracy of 0.734, indicating moderate discriminative ability across individuals. In detail, class-specific metrics showed high sensitivity and precision for several donors, particularly Stallion 9, which was nearly perfectly classified (Sensitivity = 1, Precision = 1, F1 ≤ 0.923). Donors 3 and 5 also showed strong classification metrics (F1 ranging from 0.745 to 0.923), whereas stallions 7, 8, and 12 were less accurately classified, with low sensitivity and F1 scores (F1 ≤ 0.545), reflecting greater overlap of their feature profiles with other donors.

In contrast, the model was largely unable to discriminate among frozen–thawed sperm samples treated with different antioxidant treatments. The nested cross-validated accuracy was only 0.248, and class-specific metrics were generally low. Fresh semen showed high sensitivity (1), precision (1), and F1 score (1), whereas frozen–thawed samples, regardless of the treatment applied, exhibited very low sensitivity (<0.438) and F1 score (<0.378). This suggests that fresh semen exhibits a distinct feature profile compared with that observed in frozen–thawed samples [15]. However, the lack of discriminability among frozen–thawed sperm samples, irrespective of the antioxidant treatment applied, indicates that all treatments considered produce effect on sperm quality that are weaker than those attributable to inter-stallion variability.

A permutation test was applied to assess the statistical significance of the classification results. In this approach, the class labels of the samples were randomly shuffled, and the classification procedure (PCA followed by LDA) was repeated for 1000 permutations. This generates a null distribution of accuracies that represents the performance expected by chance.

The observed nested cross-validated accuracy was then compared against this null distribution to compute a *p*-value, defined as the proportion of permutations yielding an accuracy equal to or greater than the observed value. A low *p*-value indicates that the observed classification performance is unlikely to occur by chance, providing evidence that the model captures genuine structure in the data.

In this study, the permutation test yielded a *p*-value of 0.053 for Stallion classification, confirming the model’s significant ability to distinguish individual donors. In contrast, the *p*-value for treatment classification was 0.396, suggesting that classification based on antioxidant treatments did not differ significantly from chance.

## 4. Discussion

This study demonstrates that inter-stallion variability is the primary determinant of phenotypic differences in equine sperm quality following cryopreservation. Multivariate analysis consistently showed good discrimination by stallion identity respect to the freezing medium composition, indicating that intrinsic biological differences outweigh treatment-related effects under the conditions examined.

The analysis of three different volume variables of the spermatozoon (nucleus and midpiece) or of the entire cell as evaluated by holotomography confirms the results of our previous study [15] in which this technology was applied for the first time to evaluate qualitative characteristics of stallion spermatozoa. Also in the present study, variations in the sperm midpiece emerge as the variables most clearly influenced by environmental effects. HT was firstly conducted as a potential discriminant analysis among different freezing extenders in stallion semen [15]. In the present study, instead, the effect of adding different antioxidant substances to the freezing extender was tested. In both cases, a very marked reduction in post-acrosomial/midpiece volume was observed between fresh and frozen–thawed spermatozoa, whereas smaller differences emerged among the different groups of frozen spermatozoa. Spermatozoa frozen with the benchmark extender (CTRL+) INRA Freeze showed a significantly lower volume of the entire sperm cell compared with those in the other groups treated with antioxidants and with the control group frozen in HF-20 without added antioxidants (CTRL−). A lower volume was also observed in the post-acrosomal and midpiece region when comparing INRA Freeze and HF-20, whereas no significant differences emerged in the nuclear region, unlike what was observed in the previous study [15]. The physiological meaning of this finding is difficult to determine and highlights the need for further investigations into the possibility of using HT as a diagnostic tool for sperm quality.

The volume of the post-acrosomal and midpiece region could be interpreted as a marker of sperm quality, considering that it decreases significantly when moving from fresh to frozen spermatozoa, as also occurs for many of the sperm quality parameters analyzed. However, if we consider that in the comparison of antioxidant treatments, spirulina and quercetin showed smaller volume reductions compared to matcha and horseradish, a result not consistent with what was observed for all the other sperm quality parameters analyzed, the reliability of this potential marker requires further evaluation. The reasons for the volumetric reduction in this region during sperm freezing may be attributed to morphological changes in the components of this region (mitochondria and microtubules) as a consequence of the freezing process. Freezing procedures generate osmotic stress and progressive dehydration, leading to water efflux and contraction of cellular compartments [43,44]. In the midpiece, this process, followed by osmotic shock during thawing, can result in compaction of mitochondria and outer dense fibers, ultimately reducing the overall volume [45]. Species-specific evidence supports this interpretation. In llama sperm, cooling and freezing have been shown to induce mitochondrial vacuolization and disorganization of the midpiece, together with plasma membrane and acrosomal damage [45]. Similarly, in bull and human sperm, electron microscopy analyses have documented structural changes in the midpiece, including alterations of the outer dense fibers and mitochondrial damage [8,46]. In bulls, however, plasma membrane damage is described as more prominent, with comparatively milder mitochondrial effects. In contrast, stallion and llama sperm appear particularly susceptible to midpiece structural disruption [45,47,48]. Collectively, these observations support the interpretation that the volumetric decrease detected by holotomographic imaging reflects underlying structural impairment of the midpiece, most likely resulting from osmotic stress, dehydration, and freeze–thaw injury.

Conversely, the lack of significant variations in nuclear volume between fresh and frozen spermatozoa appears less clear. Although a significant increase in sperm DNA fragmentation following freezing was detected using both direct (TUNEL) and indirect (SCSA) methods, and in agreement with studies conducted by other authors [49,50], no variations in nuclear volume were observed in HT analysis between fresh and frozen spermatozoa. This finding would contrast with what was reported by Shin et al. [51], who, by inducing DNA fragmentation in human spermatozoa using 500 µM H_2_O_2_, described a significant volumetric change in the nuclear region. However, it should be considered that this study, reported as an abstract, does not provide detailed information on the methodologies used and is based on an experimentally rather than naturally induced event. Treatment with H_2_O_2_ may, in fact, have induced additional structural modifications in the sperm head [52] that could have influenced the outcome of the study.

The poor discriminative performance observed for extract-based classification suggests that antioxidant supplementation of the HF-20 freezing medium induces only little changes in the overall sperm phenotype that HT analysis was not able to detect. Moreover, other sperm quality assessment systems used in the present study also failed to discriminate among sperm samples subjected to freezing with different antioxidant treatments. Despite well-documented antioxidant properties, matcha, spirulina, horseradish, and quercetin produced overlapping multivariate profiles, comparable to both the basal HF-20 medium and the commercial INRA Freeze extender. These findings imply that antioxidant effects may be modest, highly donor-dependent, or insufficiently captured by the selected functional and structural parameters.

In contrast, the clear and consistent separation of fresh semen from all cryopreserved conditions confirms that freezing itself is the dominant experimental factor affecting sperm phenotype. The ability to accurately discriminate fresh samples serves as an internal positive control, validating the sensitivity of the combined PCA-LDA and holographic tomography framework. Structural data further support this interpretation, as cryopreservation selectively reduced volumes of the whole cell and post-acrosomal/midpiece regions while sparing nuclear volume, in agreement with previous reports [15].

Importantly, although spirulina and quercetin partially preserved midpiece volume, bringing these groups closer than the other frozen groups to the characteristics of fresh semen, these effects did not translate into a distinct global multivariate signature and were strongly modulated by stallion identity. This reinforces the concept that individual biological variability is a critical factor in semen cryopreservation outcomes and may mask treatment-specific effects in population-level analyses.

Taken together, these findings demonstrate that individual stallion identity constitutes the primary source of structured variation in the dataset, while the considered antioxidant supplementation to the freezing extender exerts a limited and inconsistent influence. A clear discrimination is instead observed when comparing fresh and frozen spermatozoa, regardless of the antioxidant treatment applied.

## 5. Conclusions

By integrating holographic tomography with multivariate analysis and rigorous nested cross-validation, this study provides a comprehensive assessment of equine sperm responses to cryopreservation and antioxidant supplementation. These results demonstrate that stallion identity exerts a stronger influence on sperm phenotypes than the considered antioxidant-supplemented freezing media. While the analytical framework robustly distinguished fresh from cryopreserved semen, it failed to consistently discriminate among different antioxidant treatments, indicating that their effects are modest relative to intrinsic inter-individual variability.

These findings highlight the necessity of accounting for biological heterogeneity when evaluating sperm cryopreservation strategies and caution against overinterpreting treatment effects without donor-specific analyses. Importantly, these results should not be interpreted as evidence of a lack of antioxidant efficacy; rather, they suggest that any potential benefits are subtle, context-dependent, and may be masked by donor-specific variability. Future studies incorporating molecular, metabolic, or functional endpoints, as well as personalized cryopreservation approaches, may further clarify the conditions under which antioxidant supplementation provides measurable benefits.

## Data Availability

The original contributions presented in this study are included in the article/Supplementary Materials. Further inquiries can be directed to the corresponding author.

## Supplementary Materials

The following supporting information can be downloaded at https://www.mdpi.com/article/10.3390/antiox15050642/s1: Figure S1: Classification performance of sperm samples by Stallion and Extract; Figure S2: Descriptive statistics relative to the bar plot reported in Figure 2; Figure S3: ANOVA test on volume over the different examined antioxidant extracts; Figure S4: sperm quality parameters including sperm kinetics, mitochondrial membrane potential, oxidative/nitrosative stress markers, and DNA fragmentation indices; Figure S5: PCA loading profiles for the first five principal components (PC1-PC5).

## Appendix A

Performance metrics reported in Figure 4 were computed as follows:

(A1) $$Sensitivity=\frac{TP}{\left(TP+TN\right)}\;Specificity=\frac{TN}{\left(TP+TN\right)}\;Precision=\frac{TP}{\left(TP+FP\right)}\;F1=\frac{2\cdot TP}{\left(2\cdot TP+FP+FN\right)}$$

where TP = True Positive, TN = True Negative and FP = False Positive.
