# Supplementary material for: Holotomography and Multivariate Analysis Reveal Donor-Specific Responses to Antioxidant Supplementation During Stallion Sperm Cryopreservation
Source: Antioxidants (Basel). 2026 May 18;15(5):642. doi: 10.3390/antiox15050642 (PMC13203914; doi:10.3390/antiox15050642)
Supplement: Supplementary file 1 [file antioxidants-15-00642-s001.zip › antioxidants-4291778-supplementary.pdf]

Supplementary Materials

Holotomography and Multivariate Analysis Reveal Donor-Specific Responses to Antioxidant Supplementation During Stallion Sperm Cryopreservation

Graziano Preziosi, Raffaele Boni, Stefano Cecchini Gualandi and Maria Antonietta Ferrara

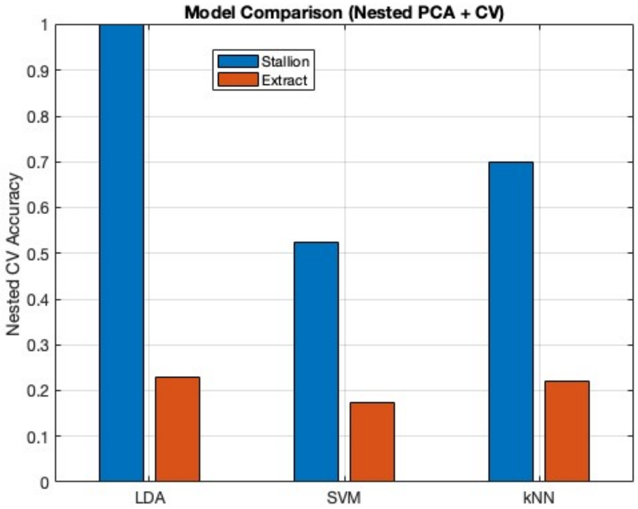

Figure S1. Classification performance of sperm samples by Stallion and Extract. (A) Cross-validated accuracy for different models (LDA, SVM, kNN) using nested PCA. Stallion-based classification (blue) outperforms Extract-based classification (orange).

(a)

Descriptive Statistics

	Volume (µm³)						
	Fresh	Matcha	Horseradish	Spirulina	Quercetin	CTRL (-)	CTRL (+)
Valid	521	513	512	515	514	509	516
Missing	0	0	0	0	0	0	0
Mean	64.666	57.603	57.870	58.457	59.023	58.818	56.115
Std. Deviation	11.415	7.721	7.060	7.118	8.154	8.728	5.762
Minimum	42.440	39.440	41.310	41.070	40.650	38.430	40.460
Maximum	101.960	100.020	91.910	79.760	85.950	98.130	76.460

(b)

Descriptive Statistics

	Volume (µm³)						
	Fresh	Matcha	Horseradish	Spirulina	Quercetin	CTRL (-)	CTRL (+)
Valid	511	506	514	512	508	512	508
Missing	0	0	0	0	0	0	0
Mean	10.638	9.720	9.685	10.096	10.097	10.031	9.690
Std. Deviation	1.988	1.773	1.679	1.644	1.865	1.834	1.488
Minimum	5.690	3.690	5.440	3.620	5.210	4.480	4.000
Maximum	17.980	15.760	15.420	15.490	15.720	18.690	14.470

(c)

Descriptive Statistics

	Volume (µm³)						
	Fresh	Matcha	Horseradish	Spirulina	Quercetin	CTRL (-)	CTRL (+)
Valid	524	520	523	523	521	522	522
Missing	0	0	0	0	0	0	0
Mean	2.485	2.461	2.374	2.565	2.555	2.532	2.410
Std. Deviation	1.641	1.527	1.598	1.435	1.628	1.517	1.493
Minimum	0.020	0.020	0.020	0.010	0.010	0.010	0.040
Maximum	14.210	7.890	9.010	7.120	9.950	8.970	8.210

Figure S2. Descriptive statistics relative to the bar plot reported in Figure 2 for whole cell region (a), post-acrosomal and midpiece region (b) and nuclear region (c) corresponding to different refractive index ranges.

(a)

Post Hoc Comparisons - Type

		Mean Difference	SE	df	t	p_{bonf}
Matcha	Horseradish	-0.267	0.467	3073	-0.571	1.000
	Spirulina	-0.854	0.467	3073	-1.831	1.000
	Quercetin	-1.420	0.467	3073	-3.041	0.036
	(CTRL (-))	-1.215	0.468	3073	-2.597	0.142
	(CTRL (+))	1.488	0.466	3073	3.190	0.022
Horseradish	Spirulina	-0.587	0.467	3073	-1.258	1.000
	Quercetin	-1.153	0.467	3073	-2.469	0.204
	(CTRL (-))	-0.948	0.468	3073	-2.026	0.643
	(CTRL (+))	1.754	0.467	3073	3.760	0.003
Spirulina	Quercetin	-0.566	0.466	3073	-1.213	1.000
	(CTRL (-))	-0.361	0.468	3073	-0.772	1.000
	(CTRL (+))	2.342	0.466	3073	5.027	< .001
Quercetin	(CTRL (-))	0.205	0.468	3073	0.437	1.000
	(CTRL (+))	2.907	0.466	3073	6.237	< .001
(CTRL (-))	(CTRL (+))	2.703	0.467	3073	5.784	< .001

Note. P-value adjusted for comparing a family of 15 estimates.

(b)

Post Hoc Comparisons - Type

		Mean Difference	SE	df	t	p_{bonf}
Matcha	Horseradish	0.036	0.108	3054	0.330	1.000
	Spirulina	-0.376	0.108	3054	-3.486	0.007
	Quercetin	-0.377	0.108	3054	-3.491	0.007
	(CTRL (-))	-0.311	0.108	3054	-2.884	0.059
	(CTRL (+))	0.030	0.108	3054	0.278	1.000
Horseradish	Spirulina	-0.411	0.107	3054	-3.831	0.002
	Quercetin	-0.412	0.108	3054	-3.835	0.002
	(CTRL (-))	-0.346	0.107	3054	-3.227	0.019
	(CTRL (+))	-0.005	0.108	3054	-0.051	1.000
Spirulina	Quercetin	-0.001	0.108	3054	-0.011	1.000
	(CTRL (-))	0.065	0.107	3054	0.604	1.000
	(CTRL (+))	0.406	0.108	3054	3.769	0.003
Quercetin	(CTRL (-))	0.066	0.108	3054	0.614	1.000
	(CTRL (+))	0.407	0.108	3054	3.773	0.002
(CTRL (-))	(CTRL (+))	0.341	0.108	3054	3.166	0.023

Note. P-value adjusted for comparing a family of 15 estimates.

(c)

Post Hoc Comparisons - Type

		Mean Difference	SE	df	t	p_{bonf}
Matcha	Horseradish	0.086	0.095	3125	0.908	1.000
	Spirulina	-0.104	0.095	3125	-1.097	1.000
	Quercetin	-0.095	0.095	3125	-0.995	1.000
	(CTRL (-))	-0.071	0.095	3125	-0.750	1.000
	(CTRL (+))	0.051	0.095	3125	0.536	1.000
Horseradish	Spirulina	-0.191	0.095	3125	-2.008	0.671
	Quercetin	-0.181	0.095	3125	-1.905	0.853
	(CTRL (-))	-0.158	0.095	3125	-1.660	1.000
	(CTRL (+))	-0.035	0.095	3125	-0.372	1.000
Spirulina	Quercetin	0.010	0.095	3125	0.101	1.000
	(CTRL (-))	0.033	0.095	3125	0.347	1.000
	(CTRL (+))	0.155	0.095	3125	1.635	1.000
Quercetin	(CTRL (-))	0.023	0.095	3125	0.245	1.000
	(CTRL (+))	0.146	0.095	3125	1.532	1.000
(CTRL (-))	(CTRL (+))	0.122	0.095	3125	1.287	1.000

Note. P-value adjusted for comparing a family of 15 estimates.

Figure S3. ANOVA test on volume over the different examined antioxidant extracts obtained for the three analyzed regions corresponding to different refractive index ranges: (a) Whole cell; (b) Mid piece; (c) Nuclear region.

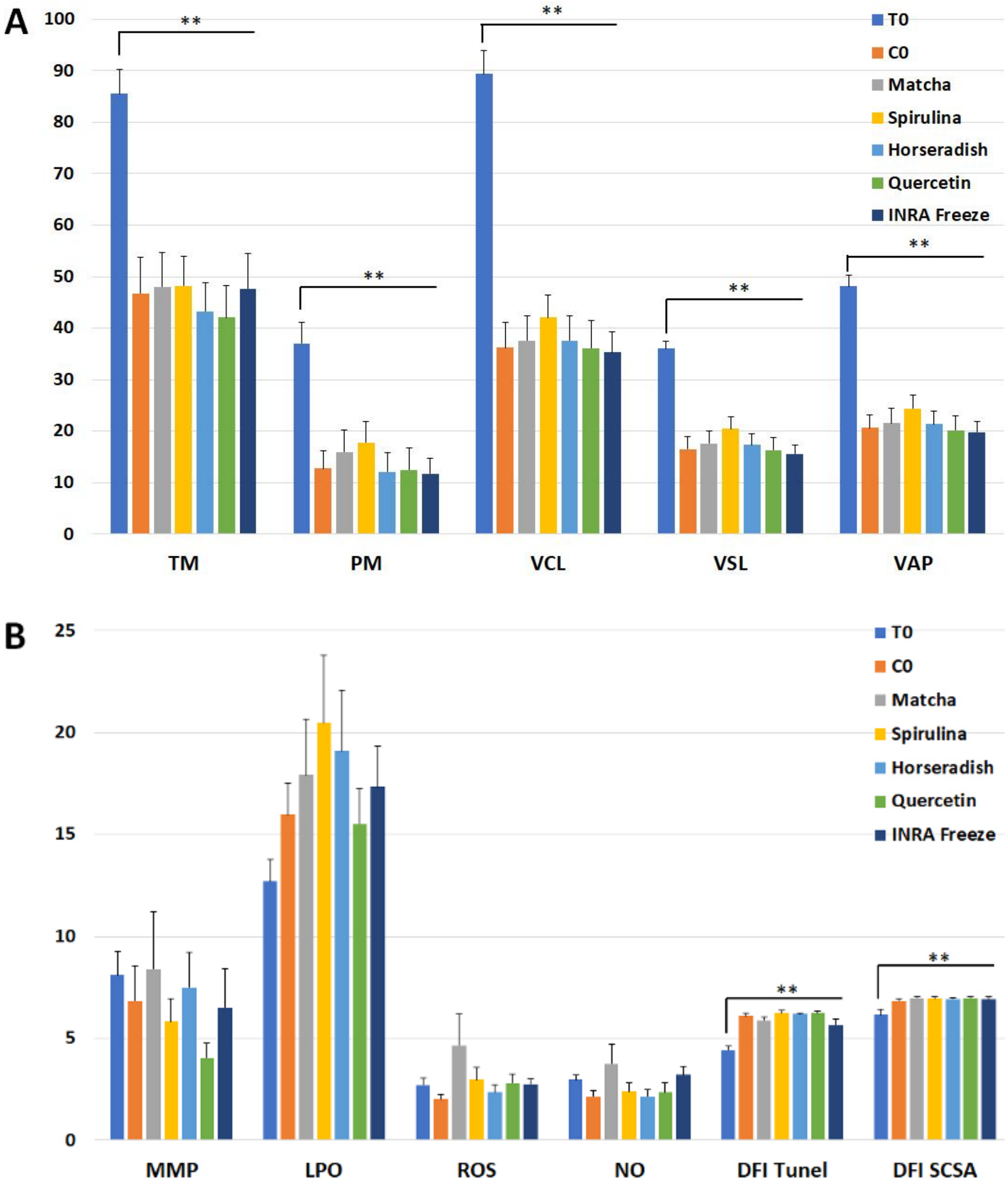

Figure S4. Sperm kinetics included total motility (TM, %), progressive motility (PM, %), curvilinear velocity (VCL, μm s^{-1}), straight-line velocity (VSL, μm s^{-1}), and average path velocity (VAP, μm s^{-1}). Sperm bioenergetics comprised mitochondrial membrane potential (MMP). Oxidative and nitrosative stress markers included lipid peroxidation (LPO), reactive oxygen species (ROS), and nitric oxide (NO). The DNA fragmentation index was evaluated using both TUNEL (DFI TUNEL) and SCSA (DFI SCSA) assays.

MMP was determined as the ratio of the second (~595 nm) to the first (~535 nm) fluorescence peak. LPO was expressed as the ratio of the first (~520 nm) peak to the sum of the first (~520 nm) and second (~590 nm) peaks. Fluorescence intensity was reported in arbitrary units (a.u.). DFI TUNEL was calculated as the ratio of green to red emission peaks, while DFI SCSA was computed as the ratio of the red peak (647 nm) to the combined red (647 nm) and green (~530 nm) peaks. ** ($p < 0.01$).

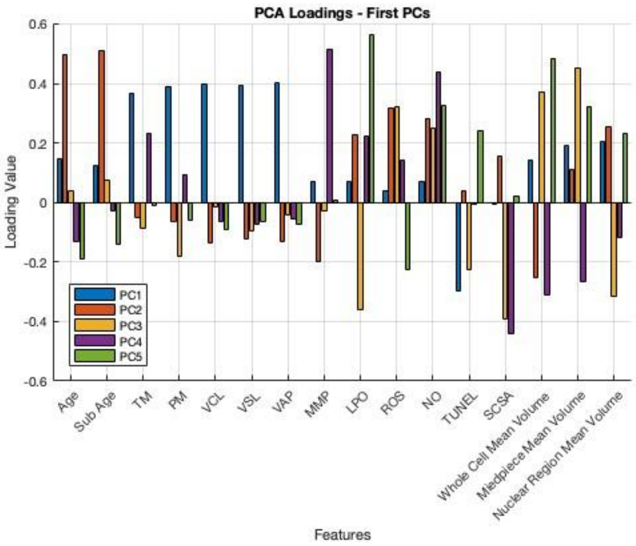

Figure S5. PCA loading profiles for the first five principal components (PC1-PC5). The plot shows the contribution (loadings) of each original variable to the corresponding principal components after z-score standardization.
